# Design of Festival Sentiment Classifier Based on Social Network

**DOI:** 10.1155/2020/8824009

**Published:** 2020-08-07

**Authors:** Huilin Yuan, Yufan Song, Jianlu Hu, Yatao Ma

**Affiliations:** ^1^College of Management, Northeastern University at Qinhuangdao, Qinhuangdao 066004, China; ^2^College of Information Science and Engineering, Northeastern University, Shenyang 110819, China; ^3^College of Computer and Communication Engineering, Northeastern University, Shenyang 110819, China

## Abstract

With the development of society, more and more attention has been paid to cultural festivals. In addition to the government's emphasis, the increasing consumption in festivals also proves that cultural festivals are playing increasingly important role in public life. Therefore, it is very vital to grasp the public festival sentiment. Text sentiment analysis is an important research content in the field of machine learning in recent years. However, at present, there are few studies on festival sentiment, and sentiment classifiers are also limited by domain or language. The Chinese text classifier is much less than the English version. This paper takes Sina Weibo as the text information carrier and Chinese festival microblogs as the research object. CHN-EDA is used to do Chinese text data augmentation, and then the traditional classifiers CNN, DNN, and naïve Bayes are compared to obtain a higher accuracy. The matching optimizer is selected, and relevant parameters are determined through experiments. This paper solves the problem of unbalanced Chinese sentiment data and establishes a more targeted festival text classifier. This festival sentiment classifier can collect public festival emotion effectively, which is beneficial for cultural inheritance and business decisions adjustment.

## 1. Introduction

In recent years, with the continuous improvement of people's living standards and the rapid development of e-commerce technology, both the growing consumption tendency in festivals and the relevant festival policies promulgated by the states show that cultural festivals are increasingly valued. Abroad, Americans spend more than 1 trillion dollars on Christmas day and English spend more than 2 billion pounds according to report from MuchNeeded. In China, Chia et al. [1] analysed the excess returns phenomenon in the Hong Kong stock market due to the pre- and post-Chinese New Year holiday effect. That investors were affected by the holiday effect in stock trading was explained with finance knowledge. Yuan and Gupta [2] studied the impact of the Chinese Lunar New Year on stock markets in six Asian countries and regions, including China, Hong Kong, and Japan, and found positive results brought by preholiday effect. According to the Consumption Trend Report on Traditional Festivals released by Freshippo, the sale growth of the four traditional festivals—Spring Festival, Tomb-Sweeping Day, Dragon Boat Festival, and Mid-Autumn Festival—is far higher than that of foreign festivals in China, with a sale increase of more than 20% in 2019. Chinese government has also published the opinions on the implementation of the inheritance and development project of Chinese traditional culture in 2017. All the facts have proved that the festivals affect peoples' lives in almost every aspect. Therefore, if the public festival emotions can be grasped correctly, reasonable emotional analysis is conducive to a better cultural development and economic decisions.

Nowadays, as a tool for communication and entertainment, social platforms are frequently used. For example, Facebook, a large and well-known social network, has about 215 million unique visitors in the United States and more than 2 billion users. Twitter reported 126 million daily users in 2018. Due to the ample functions of social media such as retweets, comments, and thumbs up, it is more possible for an individual to be influenced by others and show their thoughts, viewpoints, and attitudes on the social platform [3, 4]. Hence, these social texts gradually become suitable resource for data mining and text analysis. Many valuable studies about sentiment analysis and prediction are done based on data mined from social media [5–9].

Text mining by using social networks data has been studied for several years. Researchers studied on identity [10], lexicon [11, 12], and social relationship [13] based on social network information. Some customer opinions [14] and other short texts [15, 16] on social software are often used as information sources to study emotion classification. However, due to the limitations of language structure and data type, Chinese text classifiers are less studied than the English version, and the accuracy of classifiers in some specific fields cannot be guaranteed. Through the processing of data, the comparison of classifiers, and the determination of parameters, a Chinese festival sentiment classifier with good prediction accuracy can be established in this paper. In the context of the current worldwide, a better understanding of the public's emotional tendency and the development trend of public opinion on traditional festivals is beneficial for the country to let the public better inherit and carry forward the traditional culture of the nation. For businesses, it is helpful to make the right decision, so as to improve their own greater benefits with less cost.

This paper is organized as follows. Related work is described in Section 2. And, then the methodology, including CHN-EDA, CNN, DNN, and naïve Bayes, used in this paper is presented. An experiment toward microblo data mining and analysis is shown in Section 4.  Section 5 presents a conclusion.

## 2. Related Work

Bi et al. [17] proposed a methodology related to product/service attributes for conducting IPA (importance-performance analysis) based on sentiment strengths of online reviews. And, more effective analysis results could be obtained by the proposed methodology with lower cost and shorter time. Lin et al. [18] proposed opinion-mined prototype based a tree-like retweeting structure to cope with the evolution of the microblog opinion. Sentiment was classified by an analysis method based microblog sentiment lexicon. Liu et al. [19] focused more on the subjective preference of the consumer and proposed an algorithm based on sentiment dictionaries to identify the positive, neutral, or negative sentiment orientation of online reviews. Feng et al. [20] proposed a novel hierarchial-structure LSTM model with context attention to do sentiment classification. Jiang et al. [21] used a six-dimensional vector, to compute sentiment of news event based on Weibo. In previous sentiment analysis work, short texts on social network are usually divided into texts with positive emotion and negative emotion. However, only positive and negative two polarity emotions are not enough to classify exactly the sentiment of the public in this study. Finally, data in this paper are manually classified into 4 emotion categories.

Deep neural network (DNN) has been used in many practical data analysis models [22, 23]. Dos Santos and Gatti [24] denoted to do binary sentiment classification on short texts by a deep convolutional neural network (CNN) based on character- to sentence-level information. They achieved higher prediction accuracy on single sentence sentiment prediction. A new neural network approach proposed by Tang et al. [25], incorporating user-level and product-level information, worked for sentiment classification. Bi et al. [26] proposed an ensemble neural network-based model (ENNM), which could analyse complex relationships among factors obtained from online reviews, to measure the effects of customer sentiments toward different customer satisfaction dimensions (CSDs) on customer satisfaction. An effect-based Kano model (EKM) was proposed and used to classify the CSDs into different categories. Ibrahim and Yusoff [27] classified tweets into three categories by naïve Bayes classifier based on trainers' perception in order to improve the accuracy of text classification for sentiment analysis.

All in all, sentiment analysis on short texts based social networks is still popular topic in recent years. Considering Twitter and Facebook is not used commonly in China, this paper chooses social network Sina Weibo as text analysis platform. Referring to papers, this paper decides to compare different commonly-used methods, including CNN, DNN, and naïve Bayes to establish a more accurate festival classifier.

## 3. Materials and Methods

### 3.1. Data Acquisition

#### 3.1.1. Platform Selection

**Figure 1:**
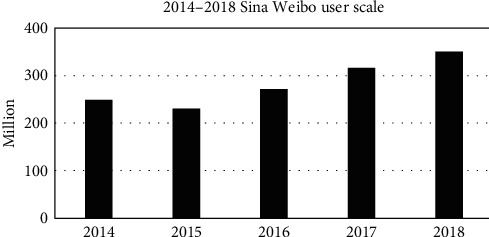
Sina Weibo user scale from 2014 to 2018.

In China, Weibo has gradually become an important part in people's daily life since Sina launched this service in 2009. Increasing number of people use this social platform to participate in the discussion of hot social topics. According to China Internet Network Information Centre (CNNIC), the user scale of Sina Weibo from 2014 to 2018 is obtained. As can be seen from the Figure 1, the user scale shows a continuous increase tendency with time going on, from 230 million to 351 million.

On one hand, Weibo achieves the integration of individuals and society through photos, short videos, retweets, comments and other functions. As a representative of Chinese new network media, Weibo provides platform for users to post actual emotions and opinions and make friends. On the other hand, the openness, interactivity, terminal scalability, operation simplicity, and big user scale make Sina Weibo quickly become one of the most important social media [5]. Therefore, a large amount of valuable research data is created by Sina Weibo. That is why Weibo is selected for this study.

#### 3.1.2. Data Preprocessing

Python language is suitable to simulate functions such as microblog login and advanced search to write crawler program [28], so python crawler program is used to obtain data. The date and keywords of target festivals are set as index parameters. There are a lot of dirty data, including missing, duplicate, abnormal, and invalid ones in the original data returned by the program. In order to improve the quality of the data analysis in next phase, original data are necessary to be pretreated. In this paper, it is still necessary to control that there is only one microblog posted by a same user on the same day to guarantee the accuracy of analysing the public trend. Finally, text data are saved in CSV format after the processed data are read and labelled.

### 3.2. Text Augmentation by CHN-EDA

Because of the poor result of training small data set on the performance of text categorization, Wei and Zou [29] combined with previous work on automatic text data augment, testing a number of enhancement operation derived from the computer vision [30, 31] and then proposed EDA (easy data augmentation), that is obtaining a data growth model for English text through four processes: synonym replacement, random insertion, random swap and random deletion. Since the model is only applicable to English text, this paper uses CHN-EDA by referring to other Chinese models on the forum. By adjusting the weight of synonym substitution and synonym insertion, the required data are generated in proportion. An example of CHN-EDA is shown in Table 1, which illustrates four types of text augmentation. The bold parts are the variation points. This kind of enhancement operation is equivalent to generating new textual data with similar emotions. This method is not just replication of original text, it is also effective to solve the bias of the classification models.

### 3.3. Feature Extraction-Based TF-IDF

The word frequency of the top 1000 keywords of microblogs is obtained in the feature extraction steps, and the 1*∗*1000 dimension word frequency feature is extracted by the TF-IDF method. TF-IDF reflects the importance of a word in the text, combining TF and IDF. The followings are the formulas of TF-IDF:

(1) $$\begin{array}{c} idf\left(t\right)=\log\left(\frac{n_{d}}{df\left(t\right)+1}\right),\\ tf-idf\left(t,\;d\right)=tf\left(t,d\right)*idf\left(t\right), \end{array}$$

where  *idf*(*t*) is term frequency-inverse document frequency, *n*_*d*_ means the total number of documents, *df*(*t*) denotes the number of documents which contains word *t* , and *tf*(*t* , *d*) is the frequency of the word *t*  in document *d*.

### 3.4. Deep Neural Network

**Figure 2:**
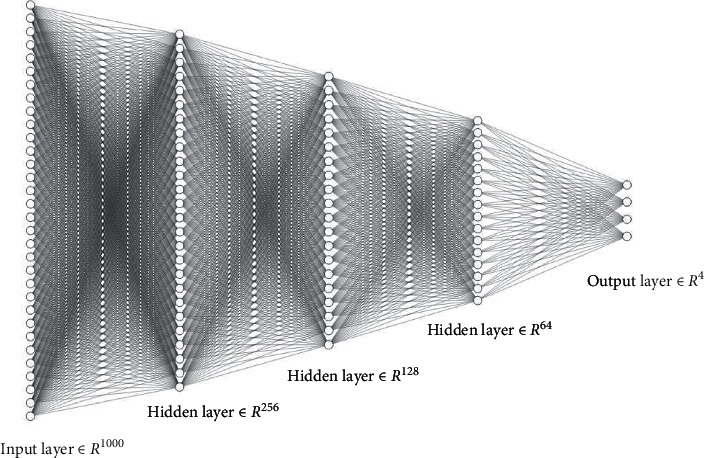
Diagram of DNN structure.

DNN structure, shown in Figure 2, consists of input layer, hidden layer, and output layer. 1^*∗*^1000 dimension word frequency feature extracted by the TF—IDF is input, and then three fully-connected layers with 256, 128, and 64 neurons, respectively, compose the hidden layer. Neuron activation function is *relu* function, with Dropout used to increase the network structure to prevent training overfitting. Because of four categories of text classified in this paper, the output layer sets four neurons, using *softmax* function as the activation function.

### 3.5. Convolutional Neural Network

**Figure 3:**
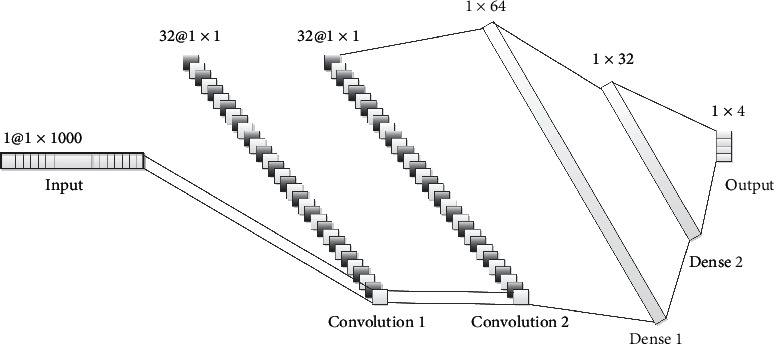
Diagram of CNN structure.

The CNN structure consists of input layer, convolution layer, fully-connected layer, and output layer, which is illustrated in Figure 3. The input layer is the 1^*∗*^1000 dimension word frequency feature extracted by TF-IDF method. In the first layer of convolution, one-dimensional convolution with 32 1^*∗*^1000 convolution kernels is used. Output of the first layer of convolution is 32 1^*∗*^1 features. The second layer convolution is a one-dimensional convolution with 32 1^*∗*^32 convolution kernels. The second layer convolution output are 32 1^*∗*^1 features. The fully connected layer contains two layers whose neurons numbers are 64 and 32, respectively, and the activation function is *relu* function. The Dropout function with a ratio of 0.5 is used. Finally, the output is divided into four categories by *softmax* function.

### 3.6. Naïve Bayes

 Naïve Bayes has different classification thought with other classification algorithms in machine learning field. It relies on calculating probability distribution to achieve classification purpose instead of directly training the relation between output class tags and input eigenvectors.

*X*(*x*_1_, *x*_2_,…, *x*_*n*_) is a input eigenvectors to be classified, and there are *m* class tags *c*_1_,*c*_2_ , … , *c_m_* as output. *P*(*c*_1_|*X*), *P*(*c*_2_|*X*),…, *P*(*c*_*m*_|*X*) is calculated to classify *X*, then the predicted category formula of *X* is

(2) $$\begin{array}{c} P\left(c_{k}\left|X\right.\right)=\max\left\{P\left(c_{1}\left|X\right.\right),P\left(c_{2}\left|X\right.\right),\ldots ,P\left(c_{m}\left|X\right.\right)\right\}. \end{array}$$

According to the conditional independence assumption of naïve Bayes, each attribute is independent from each other. The conditional probability expression is as follows [32]:

(3) $$\begin{array}{c} P\left(C\left|X\right.\right)=\frac{P\left(X\left|C\right.\right)P\left(C\right)}{P\left(X\right)}. \end{array}$$

Take the comment “Happy Lantern Festival” as an example [28]:

(4) $$\begin{array}{c} P\left(\text{Happy, Latern Festival}\left|\text{Joy}\right.\right)=P\left(\text{Happy}\left|\text{Joy}\right.\right)*P\left(\text{Latern Festival}\left|\text{Joy}\right.\right)\\ P\left(\left.\text{Joy}\right|\text{Happy, Latern Festival}\right)=\frac{P\left(\text{Happy, Latern Festival}\left|\text{Joy}\right.\right)*P\left(\text{Joy}\right)}{P\left(\text{Happy, Latern Festival}\right)}\\ \;=\frac{P\left(\text{Happy}\left|\text{Joy}\right.\right)*P\left(\text{Latern Festival}\left|\text{Joy}\right.\right)*P\left(\text{Joy}\right)}{P\left(\text{Happy},\text{Latern Festival}\right)}. \end{array}$$

## 4. Experiment Results and Discussion

### 4.1. Evaluation Indicators

The commonly used evaluation indicator [33], precision (*P*), recall (*R*), and *F*_1_ score, is set to evaluate the proposed classifier in this paper. The parameters are shown in Table 2 and the formulas are calculated as

(5) $$\begin{array}{c} P=\frac{a}{a+b},\\ R=\frac{a}{a+c},\\ F_{1}=\frac{2\times P\times R}{P+R}. \end{array}$$

It is worth mentioning that, although these indicators are commonly used in the evaluation of binary classifier, it is also suitable to evaluate the effect of different class labels in such study [34]. To have a comprehensive consideration, accuracy (A) is also added into evaluation.

### 4.2. Data Set

#### 4.2.1. Original Data from Crawler

Because this paper chooses traditional Chinese festivals as the objects of study, the crawler program is conducted to capture the data in the Dragon Boat Festival, Lantern Festival, Tomb-Sweeping Day, and Mid-Autumn Festival for five years (from 2014 to 2018). The program collects the data by setting the date and key words of the four festivals. In order to ensure the validity of data and the reliability of analysis, the size of data in different festivals needs to be in balance. In total, there are 29574 microblogs crawled from Sina Weibo as experimental text data. An example of the related data obtained by crawlers is shown in Table 3. The collected dimensions contain date, microblog ID, username, user title (including Weibo membership, Weibo personal authentication, Weibo official authentication, Weibo talent, and none), microblogs, number of retweets, and number of comments.

#### 4.2.2. Manual Label

The traditional sentiment classifier is to perform the binary classification of the texts, that is, the emotion of a text is positive or negative. However, this kind of classifier is not enough to know exactly the sentiment of the public in some situations. In this paper, 29574 microblogs are manually classified into 4 emotion categories in which emotions are divided as joy, angry, bored, and sad. Numbers of data with 4 different labels are shown in Table 4, including 26059 microblogs with *joy* emotion, 233 ones with *angry* emotion, 121 *bored* ones, and 3161 *sad* ones. Information in Table 5 illustrates examples of data with manual labels.

#### 4.2.3. Process of CHN-EDA

Based on the results of the manual label of the data, there is a serious imbalance in the data. For example, there are 26059 microblogs belonging to *joy* category, but only 3161 ones belonging to *sad* and 122 belonging to *bored*. Such question above can lead to problems like overfitting, which then results in a decrease in the accuracy of the classifier. It can be seen from Table 6 that only *joy* category has a greater than 90% precision. Precision, recall, and *F*_1_ score of data with *angry* label are just 19%, 15%, and 17%, respectively. The *F*_1_ score of *sad* label is 51%, which is a little better than *bored* label whose *F*_1_ score is just 11%. According to existing literatures [34], some relative solution, such as resampling technique which is just duplicating texts, cannot guarantee the quality of the data. The effect of backtranslation technique [35] depends on the quality of translation. Syntax, sentence extension, and contraction produce sentences with similar structure to the original sentence, but this operation is not easy to achieve and may result in the loss of semantic information. Finally, the CHN-EDA-based on EDA technique [29] is used to address the imbalance issue after comparing some currently applied methods' advantage and disadvantages.

Remarkable results are shown in Table 7 after using CHN-EDA text augmentation. For *joy* label, the improvement of classification effect is not obvious after CHN-EDA processing. This is because that too much *joy*-labelled data in the original data set result in 88% data with *joy* label in the test and training sets, which leads to the bias of the model. In other words, if a new data is predicted, the model is more inclined to classify it as a *joy* label. It is true that there is indeed more *joy* label data, so it will probably be shown that the classification accuracy of the label is very high, but it does not represent the actual prediction accuracy. Among the remaining three labels, *sad* increases the label precision, recall rate, and *F*_1_ by 25%, 42%, and 33%, although the number is not as much as that of *angry* and *bored* whose rates rise by nearly 80%. The reason is that *sad* label data is the second most data label in original data set, so its prediction is also influenced much due to the bias impression of the model. Overall, what can be learned from the results is that it is necessary for the original data set to be processed by CHN-EDA.

### 4.3. Festival Classifier Establishment

#### 4.3.1. Sentiment Analysis Method Comparison

Traditional sentiment analysis methods, including CNN, DNN, and naïve Bayes, are compared to establish more efficient sentiment classifier. The accuracy and average performance (the average accuracy of different labels classification) of different classifiers are tested before and after using CHN-EDA data augmentation. As can be seen from Table 8, on one hand, better results can be obtained by all the algorithms after the text augmentation. Under the CNN method, the overall classification accuracy of the classifier is improved by nearly 3%. The classifier accuracy increases from 89.61% to 93.76% with the DNN method. Although the classification effect of naïve Bayes is not ideal, it still could be seen that the classification accuracy is significantly improved after EDA data augmentation. The above experimental results also prove the necessity and effectiveness of using CHN-EDA in this festival classifier. On the other hand, the traditional naïve Bayes classifier shows lower accuracy and the average performance is also lower than that of CNN and DNN classifiers, which indicates that the classification of different labels is not accurate enough, so naïve Bayes is not taken into account in the following. Combined with the selection of the optimizer, CNN and DNN methods will be compared further later.

#### 4.3.2. Sentiment Analysis Algorithm Complexity Comparison

The time complexity of neural network algorithm is related to many variables such as epochs, dataset size, number of layers, and convolution kernel size and requires intralayer multiplication and interlayer accumulation, making the calculation more complex. So, it is not easy to compare the neural network complexity directly because it is hard to use a simple formula or mathematical expression to represent it accurately. However, since small changes in each variable can cause changes in the calculation time, running time of the algorithms can reflect the algorithm complexity to a certain extent. Thus, the algorithm complexity is compared in this paper by comparing the running time of the algorithms.

On the basis of comparing the accuracy of CNN and DNN algorithm, this section verifies the running rate of the two algorithms. To compare the algorithm complexity, the models use python3 to execute the tensorflow framework on a win10 system computer with an i7-8700k 3.7 GHz Intel core CPU, 32 GB of memory, and GTX2080 8 GB GPU. The running time of CNN and DNN algorithm is tested under the same conditions. The experiment runs for a total of 10 times to reduce the uncertainty during operation, and the results of the 10 times are averaged as the final result, as shown in Table 9. The running time of the algorithm is measured in seconds.

By comparing the running time of the two algorithms, it can be seen that the average running time of CNN algorithm with 10 tests is 37.803 seconds, while the average running time of DNN algorithm is 33.187 seconds. DNN algorithm is superior to CNN algorithm in time performance with obvious advantages.

#### 4.3.3. Optimizer Selection

In the process of constructing the optimal classifier, the optimizer with better matching effect needs to be selected. Although some experimental results show that many optimizers have good optimization effect, it is hard to find the optimal one objectively. Combined with the specific problems studied in this paper, the more suitable optimizer and the optimal matching parameters are confirmed through relevant experiments.

**Figure 4:**
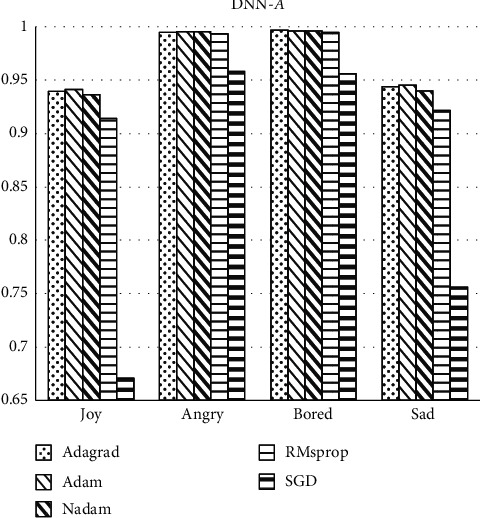
Accuracy of labels classification under the DNN method.

**Figure 5:**
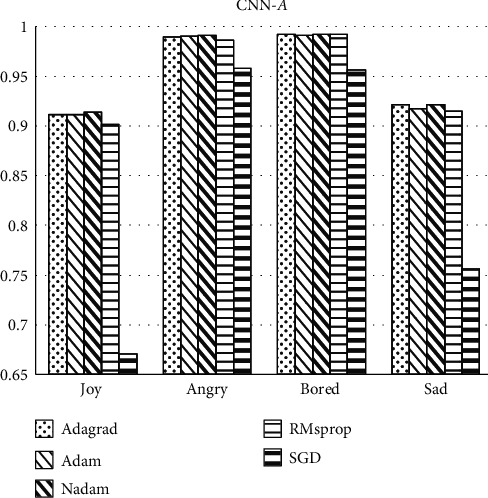
Accuracy of labels classification under the CNN method.

Firstly, six optimizers that are widely used in the field of machine learning, namely, Adagrad, Adam, Nadam, RMsprop, and SGD are selected. With the classification accuracy of the four labels as the evaluation index of this section, the effect of the classifier is compared under DNN and CNN methods, respectively. It can be seen from Figures 4 and 5 that the SGD optimizer has obvious differences and disadvantages from the other four optimizations. The classification accuracy of *joy* label with SGD optimizer is just 67%, which is much lower than other optimizers with more than 90% accuracy. And, models with SGD optimizer show 75% accuracy in *sad* label while other optimizers have accuracy about 93%. So, the SGD optimizer is not used as the research object in the following experiments. The classification accuracy of labels cannot clearly reflect the significant difference in the optimization effect of the remaining optimizer. Therefore, *F*_1_ score is then used as the baseline for optimizer comparison, because this indicator considers both precision rate and recall rate, so as to get a more accurate and objective comparison conclusion.

**Figure 6:**
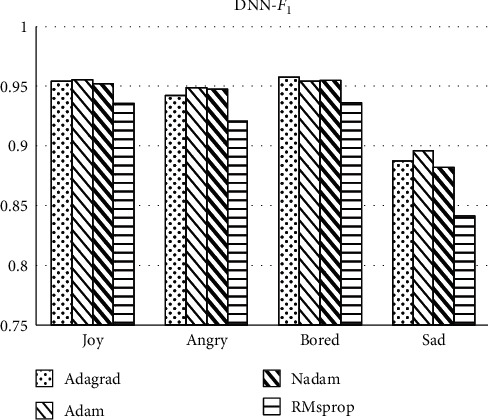
*F*_1_ score of labels classification under the DNN method.

**Figure 7:**
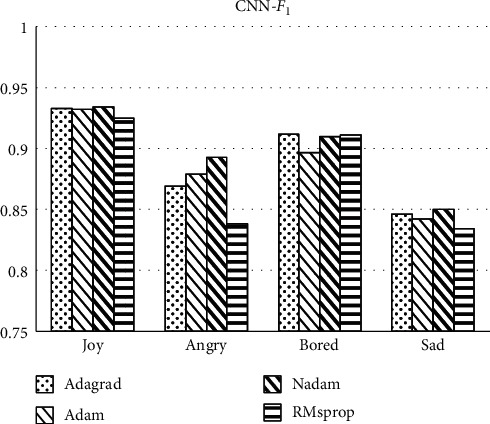
*F*_1_ score of labels classification under the CNN method.

Comparison between Figures 6 and 7 shows *F*_1_ scores of most labels' classification under the DNN method are about 95% which is higher than that of labels under the CNN method. The scores in *angry* label under CNN are lower than 90%. The indicators are even less than 85% in *sad* label. Above all, *F*_1_ score comparison results show that firstly, the overall operation effect of DNN is higher than that of CNN. Secondly, in terms of optimization ability of the optimizer, Adagrad is more suitable for the optimization algorithm of this classifier.

#### 4.3.4. Learning Rate of the Optimizer

**Figure 8:**
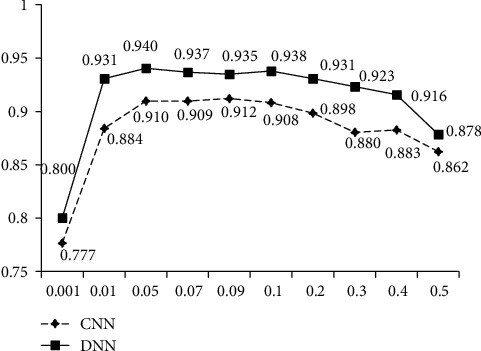
Accuracy of classifiers with different learning rates.

This section determines the final learning rate of the optimizer through experiments. The results in Figure 8 show, when the learning rate is 0.05, the accuracy of DNN sentiment analysis classifier reaches the highest point, that is, 94%. From Figure 8, it can also be found that, in this environment, the accuracy of DNN method is always higher than that of CNN. Combined with the above experiments, it can be concluded that the DNN algorithm is more suitable for this study. DNN is finally identified as the festival sentiment classifier and Adagrad as the matching optimizer.

## 5. Conclusions

Through experiments, after comparing different text analysis methods, CNN, DNN, and naïve Bayes, the method and parameter values for festival classifier can be determined finally. Experiment results illustrate DNN as sentiment analysis model and the Adagrad optimizer with a learning rate of 0.05 are suitable to build such a classifier.

The major contribution of this paper is that this paper combines the festival data and sentiment analysis models, establishing a Chinese festival classifier under the traditional festivals background, which can obtain public's festival sentiment effectively. Moreover, CHN-EDA is applied for solving the bias of the model caused by sentiment microblog imbalance. Through changing weight of synonym substitution and synonym insertion, a suitable dataset is generated in proportion automatically.

In the future research, more analytical methods can be compared to explore a more efficient classification model. And, such thought can be applied to the business decision and festival sentiment trend analysis, in order to play its greater practical value.

## Figures and Tables

**Table 1:** Example of Chinese text augmentation by CHN-EDA.

Operation	Sentence
Original	清明找雨神 生无可恋的扫墓又塞车又热
	To find rain god on tomb-sweeping day. I had nothing left to live for with traffic jam and hot.

Synonym replacement	清明找雨神 生无可恋的扫墓又塞车又**高热**
	To find rain god on tomb-sweeping day. I had nothing left to live for with traffic jam and heat.

Random insertion	清明**难免**找雨神 生无可恋的扫墓又塞车又热
	To unavoidably find rain god on tomb-sweeping day. I had nothing left to live for with traffic jam and hot.

Random swap	找**清明**雨神 生无可恋的扫墓又塞车又热
	To find tomb-sweeping day rain god. I had nothing left to live for with traffic jam and hot.

Random deletion	找雨神 生无可恋的扫墓又塞车又热
	To find rain god. I had nothing left to live for with traffic jam and hot.

**Table 2:** Parameters in evaluation.

	Certain class label	Other class labels
Recognized as certain class label by the classifier	*a*	*b*
Recognized as other class labels by the classifier	*c*	*d*

**Table 3:** Example of the Weibo information obtained by the crawler.

Date	2018-3-2
Microblog ID	4213002779060242
Username	好姑娘来自北方 (good girl from northern)
User title	微博会员 (Weibo membership)
Microblogs	好友早安 元宵节快乐 (good morning friends happy lantern festival)
Number of retweets	7004
Number of comments	509

**Table 4:** Numbers of data with different labels after manual labelled.

Dataset	Joy	Angry	Bored	Sad
29574	26059	233	121	3161

**Table 5:** Examples of data with different labels.

Joy	Angry	Bored	Sad
早上好, 端午节快乐∼(good morning, happy dragon boat festival ∼)	好好的元宵节 遛个狗也能被气死 (walking a dog can be angry to death in the lantern festival.)	最讨厌的是过节! 讨厌鞭炮声 (hate festivals mostly! hate the sound of firecrackers.)	悲剧的中秋节 (a tragic mid-autumn festival)

中秋快乐, 一家团圆 (happy mid-autumn festival, a family reunion.)	今年的端午节礼物, 火车晚点三个小时!! (dragon boat festival gift this year, the train delays three hours!!)	中秋节 坚决不吃月饼 恶心 (mooncakes are definitely not eaten on mid-autumn festival. Disgusting)	2016年的清明节, 心痛的无法呼吸…… (2016 tomb-sweeping day, too much heartache to breath.......)

**Table 6:** Original emotion classification results.

Emotion category	Precision	Recall	*F*_1_
Joy	0.93	0.95	0.94
Angry	0.19	0.15	0.17
Bored	0.10	0.12	0.11
Sad	0.54	0.48	0.51

**Table 7:** Emotion classification results after using CHN-EDA.

Emotion category	Precision	Recall	*F*_1_
Joy	0.95	0.91	0.93
Angry	0.93	0.83	0.88
Bored	0.92	0.87	0.90
Sad	0.79	0.90	0.84

**Table 8:** Performance under different models.

Model	Accuracy	Average performance
CNN	0.8797	0.9398
+CHN-EDA	0.9073	0.9536
DNN	0.8961	0.9480
+CHN-EDA	0.9376	0.9688
Naïve Bayes	0.4645	0.9404
+CHN-EDA	0.5247	0.8352

**Table 9:** Comparison of algorithms in running time.

Number of runs	Algorithm run time		Number of runs	Algorithm run time	
	CNN	DNN		CNN	DNN
1	37.25	33.50	6	38.35	33.40
2	36.92	33.95	7	37.67	33.04
3	37.17	33.51	8	38.46	32.69
4	37.08	33.49	9	38.69	32.16
5	37.91	33.46	10	38.53	32.67

Average run time	37.803	33.187			

## Data Availability

Original social text data are obtained by Python crawler programming, which is operated by setting the date and keyword as index parameters of the microblogs. All the data used in this paper are collected from the Sina Weibo (https://weibo.com), one of the most popular social networks in China.
